# Asymptomatic Malaria in Households and Neighbors of Laboratory Confirmed Cases in Raya Kobo District, Northeast Ethiopia

**DOI:** 10.4314/ejhs.v32i3.19

**Published:** 2022-05

**Authors:** Yimer Melese, Megbaru Alemu, Mulat Yimer, Banchamlak Tegegne, Tigist Tadele

**Affiliations:** 1 Dessie Health Science College, Ethiopia; 2 Department of Medical Laboratory Science, College of Medicine and Health Sciences, Bahir Dar University, Ethiopia; 3 Amhara Public Health Institute, Bahir Dar, Ethiopia; 4 Department of Biomedical Sciences, College of Medicine and Health Sciences, Bahir Dar University, Ethiopia

**Keywords:** Asymptomatic malaria, Reactive, Raya, Ethiopia

## Abstract

**Background:**

Malaria is the leading vector-borne parasitic disease that is causing high morbidity and mortality worldwide. So far huge efforts to control and eliminate malaria are hindered by the occurrence of asymptomatic carriers that are a potential source of infection. Yet, there is a scarcity of data nationally and in the current study area as well. Therefore, this study was aimed to assess the prevalence of asymptomatic malaria in Northeast Ethiopia.

**Methods:**

A community-based cross-sectional study was conducted in 2019 involving a total of 270 study participants recruited via purposive non-probability sampling technique. A structured questionnaire was used to collect data on sociodemographic characteristics, individual and household factors related to asymptomatic malaria. Data were entered in Epi Data 3.1 version and analyzed by using SPSS version 20, and p< 0.05 was considered statistically significant.

**Results:**

The overall prevalence of asymptomatic malaria was 7.0%, with 3.0%, 5.2%, and 12.0%, respectively by Rapid diagnostic tests (RDT), Microscopy and Polymerase chain reaction (PCR). The majority of infections (73.7%) were identified from index households. Previous malaria history (AOR: 4.030, 95% CI: 1.021–15.903), living with index cases (AOR: 3.880, 95% CI: 1.275–11.806) and family size > 6 members (AOR: 4.820, 95% CI: 1.260–18.437) were significant predictors of asymptomatic malaria.

**Conclusion:**

Reactive case detection had identified considerably higher asymptomatic malaria cases in the community. Therefore, active case investigation should be established in the community by tracking the symptomatic cases at the health facilities.

## Introduction

Malaria is a life-threatening vector-borne disease caused by a protozoan parasite of the genus *Plasmodium*, and transmitted through the bite of infected female Anopheles mosquitoes. Malaria is a serious public health problem in many parts of the world, exacting an unacceptable toll on the health and economic welfare of the world's poorest communities. Based on the WHO report, there were an estimated 228 million malaria episodes, and 405 000 malaria deaths in 2020, of which approximately 90% of the burden resided in Africa. Approximately 90 % of malaria deaths occurred in children under 5 years of age and pregnant women (1). In Ethiopia, nearly 75% of the country's landmass where 68% of the population lives is potentially malarious. Overall, malaria accounts for approximately 5% of outpatient consultations, 2.4% of admissions, and 2.5% of inpatient deaths in the country. *Plasmodium falciparum* and *Plasmodium vivax* are the two predominant species distributed all over the country, accounting for 60% and 40% of malaria cases, respectively. The main malaria control strategies in Ethiopia include but not limited to early diagnosis and prompt treatment, vector control, epidemic management, and personal protection through the use of insecticide-treated nets (2).

Scale-up of malaria laboratory diagnostics have given better opportunities to improve early detection of the parasite and treatment, which is the pillar of malaria elimination. Malaria microscopy and Rapid diagnostic tests (RDTs) are the most widely used diagnostic methods for clinical malaria and routine surveillance (3). However, low-density infections tend to be missed by these methods, necessitating the use of nucleic acid amplification-based and loop-mediated isothermal amplification methods which showed tremendous improvements in the investigation of asymptomatic malaria (4,5).

Malaria control activities have substantially reduced malaria incidence over the last decade through different strategic interventions, including environmental management, indoor residual spraying, long-lasting insecticide-treated nets, and early diagnosis and prompt treatment (6). Ethiopia has achieved a 50% reduction of malaria cases and deaths with a scale-up of malaria interventions (7). As malaria transmission declines, the distribution increasingly turned out to be focal to certain geographical areas and demographic groups (8,9).

The presence of high prevalence asymptomatic carriage poses a threat to malaria elimination and control programs (10). Since asymptomatic infection presents no overt signs and symptoms, they do not get clinical attention. Moreover, asymptomatic cases are a potential source of infection in high and low transmission settings (11).

The longtime asymptomatic carriage potentially bridges during lengthy dry season until the next transmission time, where *Anopheles* mosquitoes increase in number by harboring the gametocyte stage to continue the cycle (12). Although the scale-up of malaria interventions resulted in the subsequent reduction of malaria cases, the program demands the adoption of new strategies and the introduction of new activities for the transition of malaria control to the elimination phase (13). To interrupt the ongoing malaria transmission in a moderate and low endemic setting, the malaria elimination program needs to target the detection of asymptomatic infection in the general population through active rather than passive case detection (14).

Despite the presence of some pocket data, the prevalence of asymptomatic carriage has not been yet known nationwide and in the study area in particular. Conventionally, Ethiopia has used passive case detection and only catches up symptomatic cases who seek diagnosis and treatment after visiting the health facility (2). Therefore, the aim of this study was to assess asymptomatic malaria in households and neighbors of laboratory-confirmed cases in the catchment *kebele*s of two selected health centers in Raya Kobo District.

## Materials and Methods

**Study design, period, and area**: This cross-sectional study was conducted in the catchment *kebeles* of two selected health centers in Raya Kobo District, Northeast Ethiopia from February to April 2019. The altitude of the area ranges from 1,100 to 3,000 meters above sea level. The mean monthly temperature and the mean annual rainfall of the study area range, respectively from 19.5°C -26.21° and 411.51 mm - 1,651mm. The district is one of the country's malaria-endemic areas, and the major and minor malaria transmission seasons occur from September to December and April to May, respectively (15).

According to the 2007 census conducted by the Central Statistical Agency (CSA) of Ethiopia, the district had a total of 221,958 inhabitants (111,605 male, 110353 female) (16). The district has 37 rural and 5 urban *kebeles*. There was one hospital, seven health centers, and forty-two health posts.

**Study participants**: The study participants for this particular research work were household contacts and neighbors of laboratory-confirmed malaria cases that lived at least for a year in the area. However, children below 6 months, individuals who had taken anti-malaria drugs within 4 weeks prior to data collection, and members of households and neighbors of laboratory-confirmed malaria cases with clinical signs and symptoms of malaria (fever, chills, a nd rigor) were excluded.

**Sample size and sampling technique**: A sample size of 270 was calculated using single population proportion statistical formula; by the following assumptions: 95% level of confidence, 3.5 % margin of error and P (proportion) of 95 % taken from a previous study (17).

First, two health centers were purposely selected from Raya Kobo district based on the 2018 monthly high malaria incidence report. Individuals who visited the selected health centers and had laboratory-confirmed malaria cases were tracked to their residence for active case investigation. Within two months period, a total of 35 index cases were found to be laboratory-confirmed malaria cases, though, only 20 cases fulfilled the inclusion criteria to initiate reactive case detection for asymptomatic malaria. All members of households and neighbors of laboratory-confirmed malaria cases within a 200 m radius were included in the study by using a convenience sampling technique (18).

**Data collection**: A structured pre-tested questionnaire was used to collect sociodemographic information individual factors (e.g., travel history and residence), and household level factors (e.g., family size, availability of mosquito nets, and wall type) related to *Plasmodium* spp infection such as residence, family size via a face-to-face interview by the principal investigator.

**Blood sample collection, handling, and transportation**: Finger prick blood specimen was collected for dried blood spot (DBS), RDT, and blood film. Three to four drops of blood were immediately spotted on Whatman 3MM filter paper for molecular analysis. The DBS was packaged individually into a double zip-lock plastic bag with desiccant and subsequently transported to Woldia General Hospital Laboratory for storage at - 20 °C. Later, it was transferred to Amhara Public Health Institute for PCR analysis.

**RDT (CareStartTM malaria Pf/Pv (HRP2/pLDH) Ag Combo Test)**: After the pouch is open for immediate use, the collected capillary sample was blotted onto the sample pad of the test kit. Approximately, two drops of buffer were added onto the buffer pad and the result was read at the end of 20 minutes.

**Blood films**: Both thin and thick blood films were prepared for the microscopic investigation of *Plasmodium* spp. The thin blood smear was fixed by carefully dropping methanol using a Pasteur pipette immediately after drying. The methanol-fixed thin smears were allowed to dry completely in the air (approximately 2 min) by placing the slides on a flat surface. The prepared blood smears were transported to the nearby health center in a slide box for Giemsa staining (19).

**Molecular Analysis (Quantitative-Real Time PCR)**

**DNA Extraction**: Briefly, three 3-mm punches of the DBS were punched out and placed into a 1.5-mL Eppendorf tube. The punches inside the Eppendorf tube were treated with ATL buffer, proteinase K, and AL buffer with brief vortex and incubation with respective addition. The mixtures were transferred into the QIAamp Mini spin column and with the subsequent addition of wash buffer AW1 and AW2. The DNA was eluted in 100 µL of elution buffer, aliquoted, and stored at -20°C until use.

**DNA amplification**: Amplification of the Genus and *P. falciparum* multiplex was performed in a 20-ml reaction tube containing 10.0 µl 2X ABI TaqMan buffer, 0.5 µl each forward and reverse primer for the Genus, 0.5 µl forward and 0.25 µl reverse primer *P. falciparum* and 5 µl of DNA template. The reactions were performed under the following cycling parameters: initial hot-start at 95° C for 15 minutes, followed by 45 cycles of denaturation at 95 °C for 20 seconds, and 45 cycles annealing at 60 °C for 40 seconds. The correct fluorescence channel was selected for each fluorescently labeled primer set and the cycle threshold values were recorded at the end of the annealing step. Any sample with a cycle threshold value of 40 or below was considered positive. Both singleplex and multiplex assays were performed for all the samples (20).

**Quality control**: The questionnaire was subject to a pre-test, and training was given for the data collectors. The RDT kits were checked for expiry dates, and positive and negative blood films were run to check the quality and integrity of Giemsa staining. As for PCR, the positive and negative controls were run accordingly to maintain quality.

**Data analysis**: The data were entered into Epi Data 3.1 version and imported to Statistical Package for Social Sciences (SPSS) version 20 (IBM, USA) for analysis. Bivariable and multivariable logistic regression was performed to assess the association between the dependent variable and independent variables. Variables with a P-value of < 0.25 in the bivariable analysis were included in the multivariable logistic regression analysis. The odds ratio was calculated with a confidence interval of 95%, and a P-value < 0.05 was considered statistically significant.

## Results

**Socio-demographic characteristics**: A total of 270 study participants residing in 60 households were enrolled in the study (72 from 20 index houses and 198 from 40 neighboring houses). The majority (61%) of the participants were female. The mean age of the participants was 21.02 (+18.6 SD) years. About 38.9%, 32.6%, and 17.0% of the participants were from Addis Kign Aradom, and Jarota (Table 1).

**Table 1:** Socio-demographic characteristics of participants in the community of selected health centers in Raya Kobo District, Northeast Ethiopia from February to April 2019 (N=270)

Variable		Frequency (%)
**Age (year)**	≤ 15	137 (50.7)
	> 15	133 (49.3)
**Sex**	Male	101(37.4)
	Female	169(62.6)
**Residency**	Urban	81(30.0)
	Rural	189(70.0)
**Living in**	Index house	72(26.7)
	Neighbors house	198(73.3)
**Family**	1 – 5 members	128(47.4)
**size**	≥ 6 members	142(52.6)
***Kebele***	Aradom	88(32.6)
	Golesha	31(11.5)
	Addis Kign	105(38.9)
	Jarota	46(17.0)

**Prevalence of asymptomatic *Plasmodium* spp infection**: The two species of *Plasmodium*; namely; *P. falciparum* 57.9% (11/19) and *P. vivax* 42.1% (8/19) were identified in this study. All the 270 blood samples collected were tested for malaria via the rapid diagnostic test (RDT) and microscopy, yielding positivity rates of 3.0% (8/270), and 5.2% (14/270), respectively. However, only 92 samples were processed by the PCR method and it gave a prevalence of malaria at 12.0% (11/92). PCR detected asymptomatic malaria about 2.7-fold and 2.3-fold higher than RDT and microscopy, respectively (Table 2).

**Table 2:** Prevalence of asymptomatic malaria by different diagnostic methods in Raya Kobo District, Northeast Ethiopia from February to April 2019 (N=270)

Result	Diagnostic methods			Overall prevalence

	RDT (n=270)	Microscopy (n=270)	PCR (n=92)	
***P. falciparum***	4	7	6	11
***P. vivax***	4	7	5	8
**Total positive (%)**	8 (3.0%)	14 (5.2%)	11 (12.0%)	19 (7.0%)
**Total negative (%)**	262 (97%)	256 (94.8%)	81 (88.0%)	251 (93%)

The prevalence of asymptomatic malaria in the index and neighbors' houses was 19.4% (14/72) and 2.5% (5/198), respectively. The highest prevalence of asymptomatic malaria was recorded at two *kebeles*; Addis Kign (10.5%) and Aradom (8.0%) (Table 3).

**Table 3:** Prevalence of asymptomatic malaria across different variables in Raya Kobo District, from February to April 2019 (N=270)

Variables		Overall prevalence % (N)
Age	<15	3.6% (5/137)
	>15	10.5% (14 /133)
Sex	Male	7.9% (8/101)
	Female	6.5% (11/169)
Household membership	Index house	19.4% (14/72)
	Neighboring house	2.5% (5/198)
Kebele	Addis Kign	10.5% (11/105)
	Aradom	8.0% (7/88)
	Golesha	0% (0/31)
	Jarota	2.2% (1/46)

**Individual and Household Factors Associated with *Plasmodium* Infection**

**Bivariable and multivariable analyses**: The bivariable analysis of individual and household factors showed a significant association with asymptomatic malaria at p-value <0.05. Consequently, age group > 15 years (COR: 3.1 [CI: 1.09–8.88]) and previous history of malaria (COR: 6.727 [1.912–23.668]) showed significant association with *Plasmodium* infection. Moreover, participants living in Addis Kign subdistrict (COR: 2.9 [CI: 1.11–7.66]), within index houses (COR: 5.5 [CI: 2.056–14.486]), and family size > 6 members (COR: 5.3 [CI: 5.291 (1.504–18.610]) were at higher odds of *Plasmodium* infection (Table 4).

**Table 4:** Bivariable and multivariable analysis of individual and household factors of *Plasmodium* infection in Raya Kobo district, from February to April 2019 (N=270)

Variables		Asymptomatic malaria		Bivariable analysis	Multivariable analysis	P- value
	
		Positive	Negative	COR (95%CI)	AOR (95%CI)	
**Age (Yrs)**	≤ 15	5	132	1	1	
	>15	14	119	3.11(1.09–8.88)	1.84 (0.55–6.24)	0.326
**HH**	Index house	12	60	5.46 (2.06–14.49)	3.88 (1.28–11.81)	0.017*
**membership**	Neighboring house	7	191	1	1	
**Family size**	≤ 5 members	3	125	1	1	
	≥ 6 members	16	126	5.29 (1.50–18.61)	4.82 (1.26–18.44)	0.022*
**Travel**	Yes	3	13	3.43 (0.89–13.29)	1.53 (0.31–7.64)	0.607
**history**	No	16	238	1		
**Malaria**	Yes	16	111	6.73 (1.91–23.67)	4.03 (1.02–15.90)	0.047*
**history**	No	3	140	1		
**Mosquitoes**	Present	17	167	1	1	
**bed net**	Absent	2	84	0.23 (0.05–1.04)	0.34 (0.06–1.88)	0.215
**Wall type**	Wood with mud/clay	16	237	0.31 (0.08–1.21)	0.27 (0.05–1.55)	0.142
	Others	3	14	1	1	
***Kebele***	Addis Kign	12	93	2.96 (1.13–7.79)	2.57 (0.85–7.8)	0.095
	Others	7	159	1	1	

Statistically significant at p-value < 0.05, HH=household, AOR= adjusted odds ratio, COR=crude odd ratio, CI= confidence interval.

Eight predictors (age, relation with index cases, *kebele*, travel history, previous malaria history, family size, wall type, and utilization of mosquito's bed net) with p ≤ 0.2 in the bivariable analysis were selected multivariate analysis to control confounder variables. After adjusting for confounders, only three variables (living with index cases, previous malaria history, and family size) showed a statistically significant association with *Plasmodium* species infection (Table 4).

The odds of *Plasmodium* infection in individuals having previous malaria history was 4.0 times higher compared to those who didn't have it. Participants who lived in the index houses were at 3.9 times higher odds of getting *Plasmodium* infection than those who lived in neighboring houses. The odds of asymptomatic *Plasmodium* infection in households who had six or more members had 4.8 times higher than families with lower members (Table 4).

## Discussion

Asymptomatic malaria becomes a serious challenge in malaria elimination. Individuals with asymptomatic malaria have low health-seeking behavior, and therefore, they remain potential reservoirs in sustaining malaria transmission (10).

In the current study, RDT detected and identified 3% of the asymptomatic *Plasmodium* spp infection. This is in line with studies in Northwest Ethiopia 4.8%, (21), Southeast Asia5% (22), Lao DPR 2.2% (23), and Haiti 1.78% (24). On the other hand, the finding of this study was found to be higher than the study done in Southwest Ethiopia with zero cases (17) and Northern Senegal with 0.4% (25). This discrepancy might be due to the parasitemia level, quality of different RDT products which may differ in detection limits.

Microscopy detected and identified 5.2% of asymptomatic infections. This is in line with studies done in Northwest Ethiopia 4.2% (21), 6.8% (26), Western Ethiopia 5% (27), and Southwest Asia 5% (22). However, this figure was lower than previous studies in Kenya 12.6% (28) and Tanzania 8% (29). Lower findings than the current study was reported in Southwest Ethiopia (17), Myanmar 1.4% (30), and Haiti with 2.8% (24). This difference might be due to the parasitemia level, variation in transmission setting, and skills of laboratory personnel.

PCR detected and identified 12% of asymptomatic *Plasmodium* spp infections. This was higher than previous studies in Ethiopia; 8.12 % (17, 18), Myanmar 2.3% (30), Zambia 8% (31). However, it was lower than findings in Southeast Asia with 20% (22), Haiti with 19.1% (24), Kenya; 20 % & 33.3% (28,32), India with 22.6% (33), and this difference might be due to variation in transmission settings, the difference in case investigation, control and preventive measure taken in each country. PCR has also invariably detected a higher proportion of asymptomatic infection with about 2.7 and 2.3 folds higher than RDT and microscopy, respectively. This finding is in agreement with reports from Brazil (34). Therefore, this substantial difference among diagnostics methods can be due to variation in the detection limit of diagnostic methods, in which PCR detects as low as 0.5 – 5 parasites /µl of blood while microscopy and RDT detect 50 and 100 parasites /µl of blood respectively (35).

This study also depicted that the predominant *Plasmodium* spp was *P. falciparum* with 57.9% and the remaining 42.1% was *P. vivax.* This finding is in line with the Ethiopian nationwide malaria figure reported by the Ministry of Health over which *P. falciparum* accounts for about 60% (2). On the contrary, other studies in Ethiopia reported *P. vivax* was the predominant species in Hadiya zone, Southern Ethiopia (36), and East Shewa zone, Central Ethiopia (37). This difference might be due to the heterogeneity of *Plasmodium* species distribution as a result of environmental and ecological changes.

Reactive case detection has identified a higher prevalence of asymptomatic infection in index houses with 19.4% compared with their neighboring houses with 3.5%. This finding is consistent with a study done in Jimma zone, Southwest Ethiopia (18). This difference likely reflects family members of index cases are with equal proximity to the breeding sites of vectors along with likely behaviors and occupations within the family. Therefore, the index cases justly denote foci of transmission to family members. This study revealed that of the total asymptomatic cases, the prevalence of asymptomatic infection varied with approximately 3 folds higher in index households compared with their neighbors within a 200 m radius.

Regarding factors associated with asymptomatic malaria infection, previous malaria history increases 4.0 times the odds of having *Plasmodium* spp infection. This finding is supported by studies done in Ethiopia (18, 21, 26). This might be due to relapse or recurrence of infection. Recurrence of infection is due to the persistence of a low level of *P. falciparum* in the circulation while relapse is due to the reactivation of hypnozoite which leads to initiation of the erythrocytic cycle in the case of *P. vivax.* However, these studies also underpinned living with index cases and family size > 6 members as potential risk factors. Reasonably, the infection is clustered in index cases' houses compared with neighbors' houses and this might be due to equal proximity to vector breeding sites or likely behaviors of family members with the index cases. As family size increases, there might be individuals who didn't have a chance to use bed nets, as universal coverage of mosquito nets is low in the country in general and the study area in particular.
